# Supplementary material for: Understanding the Transmission Dynamics of the Chikungunya Virus in Africa
Source: Pathogens. 2024 Jul 22;13(7):605. doi: 10.3390/pathogens13070605 (PMC11279734; doi:10.3390/pathogens13070605)
Supplement: Supplementary file 1 [file pathogens-13-00605-s001.zip › Supplementary Material S1. Supplementary Methods.pdf]

Materials and Methods

This study aims to comprehensively document CHIKV outbreaks, cases, and generated genomes within Africa through a literature review and secondary analysis.

2.1. Literature Review

The literature for this review was comprehensively searched on PubMed for arboviruses in Africa. To identify African genomic data, outbreaks, and cases, the review excluded studies not conducted in Africa

The PubMed search query terms:

1. "Arboviruses"[Mesh] OR "Encephalitis, Arbovirus"[Mesh] OR "Arbovirus Infections"[Mesh] OR "Chikungunya Fever"[Mesh] OR "Chikungunya virus"[Mesh] OR Arbovirus*[tiab] OR ChikV[tiab] OR Chikungunya[tiab] OR “Chikungunya fever”[tiab] OR “Chikungunya virus”[tiab] OR “Chikungunya infection”[tiab]

2. Africa[tiab] OR Algeria[tiab] OR Angola[tiab] OR Benin[tiab] OR Botswana[tiab] OR "Burkina Faso"[tiab] OR Burundi[tiab] OR "Cabo Verde"[tiab] OR Cameroon[tiab] OR "Canary Islands"[tiab] OR "Cape Verde"[tiab] OR "Central African Republic"[tiab] OR Chad[tiab] OR Comoros[tiab] OR Congo[tiab] OR "Democratic Republic of Congo"[tiab] OR DRC[tiab] OR Djibouti[tiab] OR Egypt[tiab] OR "Equatorial Guinea"[tiab] OR Eritrea[tiab] OR Eswatini[tiab] OR Ethiopia[tiab] OR Gabon[tiab] OR Gambia[tiab] OR Ghana[tiab] OR Guinea[tiab] OR "Guinea Bissau"[tiab] OR "Ivory Coast"[tiab] OR "Cote d'Ivoire"[tiab] OR Jamahiriya[tiab] OR Kenya[tiab] OR Lesotho[tiab] OR Liberia[tiab] OR Libya[tiab] OR Madagascar[tiab] OR Malawi[tiab] OR Mali[tiab] OR Mauritania[tiab] OR Mauritius[tiab] OR Mayotte[tiab] OR Morocco[tiab] OR Mozambique[tiab] OR Namibia[tiab] OR Niger OR Nigeria OR Principe OR Réunion OR Rwanda OR "Sao Tome" OR Senegal[tiab] OR Seychelles[tiab] OR "Sierra Leone"[tiab] OR Somalia[tiab] OR "South Africa"[tiab] OR “Southern Provinces”[tiab] OR "St Helena"[tiab] OR Sudan[tiab] OR Swaziland[tiab] OR Tanzania[tiab] OR Togo[tiab] OR Tunisia[tiab] OR Uganda[tiab] OR "Western Sahara"[tiab] OR Zaire[tiab] OR Zambia[tiab] OR Zimbabwe[tiab]

3. "Genetic Variation" [Mesh] OR "Genome, Viral" [Mesh] OR Genomics [Mesh] OR Phylogeny [Mesh] OR Metagenome [Mesh] OR Lineage [tiab] OR Strain [tiab] OR Genotype [tiab] OR "Molecular epidemiology" [Mesh] OR "Nucleic acids" [Mesh] OR Genetic [tiab] OR Phylogen* [tiab] OR Phylogeo* [tiab] OR Gene [tiab]

4. Search terms 1, 2 AND 3 combined

5. "Aedes" [tiab] OR "aegypti" [tiab] OR "albopictus" [tiab] Vectors [Mesh] OR “Chikungunya” [Mesh] OR “Africa” [Mesh] OR distrubtion [tiab] OR Presence[tiab] OR Prevalence [tiab]

6. "Oubreak" [Mesh] OR “Case” [Mesh] OR Lineage [tiab] OR Strain [tiab] OR Genotype [tiab] OR "epidemiology" [Mesh] “Africa” [tiab] OR “Chikungunya*Review” [tiab], “meta-analysis” [tiab]

7. "Arboviruses"[Mesh] OR "Arbovirus Infections"[Mesh] OR Arbovirus*[tiab] OR "Chikungunya virus"[Mesh] OR "Disease Surveillance"[Mesh] OR surveill*[tiab] OR monitor*[tiab] AND "Africa"[Mesh] OR "Epidemiological Monitoring"[Mesh]

Studies that met any one of the following exclusion criteria were removed:

- Experimental vector infection/competence studies
- Experimental infection studies on animals
- Insecticide resistance studies
- Vector population quantification studies - Reviews (unless meta-analyses show new estimates/results)
- Vaccine studies (Unless African genomes were produced)

Studies included:

- Outbreak and travel cases (related to travel to/from Africa) or related data
- Generation of African genomes
- Genomic and phylogenetic analyses of African genomes
- Case reports.

Additionally, countries included in this studies were; Algeria, Angola, Benin, Botswana, Burkina Faso, Burundi, Cabo Verde, Cameroon, Cape Verde, Central African Republic, Chad, Comoros, Congo, Democratic Republic of Congo, Djibouti, Egypt, Equatorial Guinea, Eritrea, Eswatini, Ethiopia, Gabon, Gambia, Ghana, Guinea, Guinea Bissau, Ivory Coast, Cote d'Ivoire, Kenya, Lesotho, Liberia, Libya, Madagascar, Malawi, Mali, Mauritania, Mauritius, Mayotte, Morocco, Mozambique, Namibia, Niger, Nigeria, Principe, Réunion, Rwanda, Sao Tome, Senegal, Seychelles, Sierra Leone, Somalia, South Africa, Sudan, Swaziland, Tanzania, Togo, Tunisia, Uganda, Western Sahara, Zaire, Zambia, Zimbabwe.

Genomic papers were reviewed in Rayyan based on the above inclusion and exclusion criteria [1]. A total of 36 (Supplementary Table S1) chikungunya genomic papers were selected for this review and followed a structured approach for data extraction and synthesis to ensure comprehensive coverage of relevant genomic studies and data.

To better interpret the transmission patterns and current burden, the formal literature review described above was supplemented with literature on vector distribution, seroprevalence, genomic surveillance studies, and case and outbreak studies and reviews.

2.2 Genomic Data

Global sequences for the study were retrieved from publicly available databases, including GenBank https://www.ncbi.nlm.nih.gov/nuccore, accessed on 15 January 2024) and the Bacterial and Viral Bioinformatics Resource Center (BV-BRC) and GISAID Epi-Arbo web interface (https://gisaid.org/ accessed on 12 January 2024 , to map genomic sequencing and surveillance efforts. A total of 7793 sequences (last accessed 12 January 2024) of partial genes and whole genomes of CHIKV were downloaded. Duplicates based on accession number were removed. This complete dataset was used to map the global genome distribution, of this 393 African genomes were then analyzed to plot the genome distribution and number of genomes produced within African countries over time. The final dataset consisted of 1570 global CHIKV sequences with >50% genome coverage, after the removal of low coverage sequences, and missing metadata such as country and date. The dataset was separated into 465 Asian genomes, 79 WA, and 1026 ECSA genomes were analyzed of which 622 sequences belonged to the IOL (accession numbers in Supplementary Table S2), after genotype classification using the Genome Detective Chikungunya Typing Tool (https://www.genomedetective.com/app/typingtool/chikungunya/).

2.3. Epidemiological Data

Outbreak and case data for CHIKV infections in Africa were obtained from the WHO-AFRO Outbreaks and Emergencies Weekly Bulletin (https://www.afro.who.int/health-topics/disease-outbreaks/outbreaks-and-other-emergencies-updates?page=1) (March 2017 to till April 2024) and outbreak and case reports obtained from literature.

2.4.Phylogenetic Analysis

Sequence datasets corresponding to each global genotype were aligned against the CHIKV reference sequence NC_00416) using Nextalign v1.3.0 alignment tool. Maximum likelihood Phylogenetic tree inference was conducted using IQ-Tree2 version 2.2.2 performed with 1000 bootstraps. Model Finder was used to choose the nucleotide substitution model for each lineage, TIM2+F+I+R3 and TIM+F+I+R2 were selected based on the Bayesian Information Criterion for the Asian and WA genotypes, respectively. Substitution models GTR+F+I+R6 were selected for the ECSA and IOL. The Molecular Clock Signal was assessed with TempEst v1.5.322 [2] and any outliers that violated the molecular clock assumption were removed. A time-scaled phylogenetic tree for each set was inferred using TreeTime v 0.10.023. The *mugration* model was applied to the time-scaled phylogenetic tree in TreeTime, after annotation of tips with their discrete sampling locations, which allows for the quantification of viral transitions between regions in Africa and globally [3].

References

1. Ouzzani, M.; Hammady, H.; Fedorowicz, Z.; Elmagarmid, A. Rayyan-a Web and Mobile App for Systematic Reviews. *Syst. Rev.* **2016**, *5*, 210. https://doi.org/10.1186/s13643-016-0384-4.
2. Padane, A.; Tegally, H.; Ramphal, Y.; Seyni, N.; Sarr, M.; Diop, M.M.; Diedhiou, C.K.; Mboup, A.; Diouf, N.D.; Souaré, A.; et al. An Emerging Clade of Chikungunya West African Genotype Discovered in Real-Time during 2023 Outbreak in Senegal. *medRxiv* **2023**. https://doi.org/10.1101/2023.11.14.23298527.
3. Wilkinson, E.; Giovanetti, M.; Tegally, H.; San, J.E.; Lessells, R.; Cuadros, D.; Martin, D.P.; Rasmussen, D.A.; Zekri, A.-R.N.; Sangare, A.K.; et al. A Year of Genomic Surveillance Reveals How the SARS-CoV-2 Pandemic Unfolded in Africa. *Science* **2021**, *374*, 423–431. https://doi.org/10.1126/science.abj4336.
